# Evidence of Exposure to USUV and WNV in Zoo Animals in France

**DOI:** 10.3390/pathogens9121005

**Published:** 2020-11-30

**Authors:** Orianne Constant, Karine Bollore, Marion Clé, Jonathan Barthelemy, Vincent Foulongne, Baptiste Chenet, David Gomis, Laurie Virolle, Serafin Gutierrez, Caroline Desmetz, Rayane Amaral Moares, Cécile Beck, Sylvie Lecollinet, Sara Salinas, Yannick Simonin

**Affiliations:** 1 Pathogenesis and Control of Chronic Infections, University of Montpellier, INSERM, EFS, 34000 Montpellier, France; orianne.constant@gmail.com (O.C.); karine.bollore@umontpellier.fr (K.B.); marion.cle@umontpellier.fr (M.C.); jonathan.barthelemy@inserm.fr (J.B.); v-foulongne@chu-montpellier.fr (V.F.); sara.salinas@inserm.fr (S.S.); 2 Parc de Lunaret—Zoo de Montpellier, 34090 Montpellier, France; Baptiste.CHENET@ville-montpellier.fr (B.C.); David.GOMIS@ville-montpellier.fr (D.G.); Laurie.VIROLLE@ville-montpellier.fr (L.V.); 3 ASTRE Research Unit, CIRAD, INRA, 34398 Montpellier, France; serafin.gutierrez@cirad.fr; 4 bBioCommunication en CardioMétabolique (BC2M), Montpellier University, 34000 Montpellier, France; caroline.desmetz@umontpellier.fr; 5 UMR 1161 Virology, ANSES, INRAE, ENVA, ANSES Animal Health Laboratory, EURL for Equine Diseases, 94704 Maisons-Alfort, France; rayane.amaralmoraes@anses.fr (R.A.M.); Cecile.BECK@anses.fr (C.B.); Sylvie.LECOLLINET@anses.fr (S.L.)

**Keywords:** usutu virus, west nile virus, flavivirus, arbovirus, seroprevalence, zoological garden

## Abstract

West Nile virus (WNV) and Usutu virus (USUV) are zoonotic arboviruses. These flaviviruses are mainly maintained in the environment through an enzootic cycle involving mosquitoes and birds. Horses and humans are incidental, dead-end hosts, but can develop severe neurological disorders. Nevertheless, there is little data regarding the involvement of other mammals in the epidemiology of these arboviruses. In this study, we performed a serosurvey to assess exposure to these viruses in captive birds and mammals in a zoo situated in the south of France, an area described for the circulation of these two viruses. A total of 411 samples comprising of 70 species were collected over 16 years from 2003 to 2019. The samples were first tested by a competitive enzyme-linked immunosorbent assay. The positive sera were then tested using virus-specific microneutralization tests against USUV and WNV. USUV seroprevalence in birds was 10 times higher than that of WNV (14.59% versus 1.46%, respectively). Among birds, greater rhea (*Rhea Americana*) and common peafowl (*Pavo cristatus*) exhibited the highest USUV seroprevalence. Infections occurred mainly between 2016–2018 corresponding to a period of high circulation of these viruses in Europe. In mammalian species, antibodies against WNV were detected in one dama gazelle (*Nanger dama*) whereas serological evidence of USUV infection was observed in several *Canidae*, especially in African wild dogs (*Lycaon pictus*). Our study helps to better understand the exposure of captive species to WNV and USUV and to identify potential host species to include in surveillance programs in zoos.

## 1. Introduction

Arboviruses are emerging pathogens on the European continent and, more generally worldwide, although they are already well established in various regions, particularly in Africa and Latin America. Among arboviruses, several flaviviruses (*Flaviviridae* family), e.g West Nile (WNV), Usutu (USUV), tick born encephalitis virus (TBEV), and Meaban (MEAV) viruses are the main flaviviruses that have been known to circulate in France [1,2,3,4]. WNV and USUV belong to the Japanese encephalitis (JEV) serocomplex in the *Flavivirus* genus and share many common features [5,6]. Their transmission cycle mainly involves ornithophilic mosquitoes such as *Culex pipiens* and birds. Wild birds play a central role in flavivirus epidemiology as they are the main amplifying hosts in the wilderness. They develop a strong and transitory viremia, are capable of infecting bird-biting mosquitoes, and may contribute to the spread of the virus with their migratory behavior [3]. Moreover, WNV and USUV occasionally infect other vertebrates including humans and horses [7]. TBEV, in the TBEV serocomplex is maintained in an enzootic cycle involving hard ticks (Ixodes ricinus in Western Europe) and rodents as the main reservoir [8]. Birds are suspected to play a role in the spread of the virus [9]. MEAV is a soft tick-borne flavivirus transmitted by *Ornithodoros maritimus* ticks among seabird colonies and has only been identified in Brittany on the Meaban island [1].

WNV was first detected in the West Nile region of Uganda in 1937 from the blood of a febrile woman [10]. Its global distribution has greatly expanded over the last two decades and is currently common in countries of Africa, the Middle East, India, Australia, Central and Southern Europe, and in North, Central, and South America where it can cause disease outbreaks [11,12,13]. Natural exposure to WNV has also been detected in a wide range of mammals (more than 100 species) [14]. USUV has gained the attention of the scientific community due to its recent incursions in Europe. It was isolated for the first time in South Africa in 1959 [15,16]. Since then, this emerging arbovirus has spread out of Africa, mainly in Europe [3]. Birds and humans infected by USUV can develop neuroinvasive pathologies (including encephalitis and meningoencephalitis). Serologic and molecular evidence of USUV presence in blood donors suggests the silent spread of this virus among asymptomatic humans [3]. Beyond birds and humans, USUV has also been detected in different mammals such as horses, dogs, wild boars, wild ruminants, bats, rodents, and shrews [17,18,19,20,21,22,23,24]. Seroepidemiological studies showed that USUV is probably endemic in several European countries. Whereas WNV is involved in occasional outbreaks or endemic in some of them, mainly in Southern Europe such as in Greece, Italy, or Spain [3,25]. Recently, increased and concomitant transmission of both viruses has been reported in Europe. Beyond a record drought and heat wave, the summer of 2018 saw the largest WNV epidemic reported in terms of countries involved. A total of 16 European countries, including France, were involved and 2083 human cases and 181 deaths were listed [26]. Similarly, the largest epizootic of USUV in Europe was observed in 2018, inducing a massive death of wild birds [3,27,28].

TBEV is the arbovirus with the highest public health impact in Europe. Thousands of TBEV neurological cases are recorded every year in Europe [29]. Human infections can occur through the bite from TBEV-infected ticks and more rarely through the consumption of unpasteurized milk or milk products from cattle, goats, or sheep. TBEV is endemic in many European countries, including the Baltic and Scandinavian countries, Slovenia, and the Czech Republic. In France, the virus has been reported in Eastern France, mainly in Alsace and since 2003 in the Alps (Haute-Savoie) and more recently in Auvergne-Rhône-Alpes (Loire and Haute-Loire) [2,30]. TBEV infections have been detected more largely in Eastern France in a serosurvey on wild boars and roe deer [31].

Monitoring viral circulation is difficult, especially for viruses with complex transmission cycles. Moreover, serological cross-reactions between flaviviruses can hamper the rapid identification of circulating viruses. Elisa tests suffer from a lack of specificity and need to be confirmed by seroneutralization assays. Zoological gardens represent sites of interest to gain knowledge about the distribution of emerging zoonotic pathogens in different animal species. Therefore, zoos have been proposed as sites adapted to the surveillance of zoonotic viruses, including WNV and USUV [32,33,34,35,36]. These sites generally contain a large diversity of captive species, including birds and mammals, and are usually close to or within urban areas. Moreover, since captive animals are regularly monitored by veterinarians, samples can be easily available. In addition, WNV and USUV have been frequently detected in birds in zoological gardens and associated with important mortality in some species [23,24,27,28]. Nevertheless, few studies have been published on WNV or USUV exposure in zoo animals [23,24,25,26]. Montpellier zoo (south of France) is close to the Camargue area, a wetland that houses a large diversity of wild bird species, including migratory birds and various mosquito populations. This environment is considered to favor the transmission of USUV and WNV [29,30,31,32]. In this study, we evaluated in priority the seropositivity against WNV and USUV in captive birds and exotic mammals from the Montpellier zoological garden. TBEV and MEAV circulation has not been evidenced in the south of France. Consequently, when positive samples of competitive enzyme linked immunoabsorbent assay (cELISA) were found negative in WNV and USUV MNTs, TBEV and MEAV virus neutralization assays were carried out to investigate infections by alternative flaviviruses. The objective was to investigate the prevalence of flavivirus infections in a large variety of species and to assess the value of integrating their monitoring within the framework of WNV and USUV surveillance programs. Our results enlarge current knowledge on mammals and birds potentially infected by these viruses and point to species with high seroprevalence as candidates for sentinel surveillance.

## 2. Results

### 2.1. Circulation of USUV/WNV in Zoo Birds

A point to note is that no obvious abnormal bird mortality was observed in Montpellier zoo. Analysis in birds were carried out on 137 specimens of 20 different species (Table 1). Using competitive enzyme linked immunoabsorbent assay (cELISA), we identified antibodies against flaviviruses in 23 out of 137 birds (16.79%, CI95%: 10.53–23.05). Among them, 20 specimens tested positive for USUV-specific antibodies by virus-specific micro neutralization test (MNT) (14.59%, CI95%: 8.686–20.51) and 2 specimens for WNV-specific antibodies (1.45%, CI95%: 0.0–3.46). Overall, our results show a greater proportion of positive specimens for USUV than for WNV. 

Moreover, one common ostrich (*Struthio camelus*) presented antibodies against an undetermined flavivirus as we did not detect TBEV or MEAVS-specific antibodies by virus-specific micro neutralization test (Table 1). The two positive birds for WNV were one orange-winged amazon (*Amazona amazonica*) and one marabou stork (*Leptoptilos crumenifer*). The individuals positive to USUV were one Abyssinian ground hornbill (*Bucorvus abyssinicus*), one scarlet ibis (*Eudocimus ruber*), and two emus (*Dromaius novaehollandiae*). We also found antibodies against USUV in 6 common peafowl (*Pavo cristatus*) and 10 greater rhea (*Rhea Americana*) representing respectively 25% and 46.7% seroprevalence in the tested populations. These species, to the best of our knowledge, have never been described as susceptible to flavivirus infection. We analyzed the samples collected from the same animals over several years in order to explore infection temporality. This analysis provided information on viral circulation over time, particularly since USUV and WNV have been only quite recently widely reported in the area. Despite the lack of a continuous sampling over years, we were able to study the temporality of infection for some of the animals. Interestingly longitudinal studies showed that all the sequentially sampled animals found seropositive on the first sampling stayed seropositive throughout the study period as all seropositive birds in 2016 were also positive in 2018 (5/5) (Table A1). Moreover, the majority of specimens were infected between 2016 to 2018 (64.2%, 9/14 for WNV or USUV-positive birds) while the remaining samples show that WNV and USUV were introduced before 2016 in the area.

### 2.2. Circulation of USUV/WNV in Zoo Mammals

The analysis of seroprevalence in mammals was performed on 274 specimens of 50 different species (Table 2). A total of 11 mammals were positive with the pan-flavivirus ELISA kit (4.01%, CI95%: 1.69–6.33). Similarly to our results with birds, most of the positives mammals had antibodies against USUV, including one Asian lion (*Panthera leo persica*), one maned wolf (*Chrysocyon brachyurus*), one Iberian wolf (*Canis lupus signatus*), and two African wild dogs (*Lycaon pictus*) (Table 2). In contrast, we detected antibodies against WNV in one dama gazelle (*Nanger dama*) (Table 2). Three animals presented antibodies against non-identified flaviviruses (*Acinonyx jubatus*, *Camelus dromedarieus*, and *Panthera leo leo*) (the corresponding sera turned out to be negative in TBEV and MEAV neutralization assays). Thus, according to the available data, only a small proportion of exotic mammals’ species was exposed to USUV or WNV infections. African wild dog has never been identified as a susceptible species to infection by USUV. To confirm the susceptibility of this Canidae to USUV infection, we analyzed the sera of 8 African wild dogs from another zoological garden (Réserve Africaine de Sigean) located 100 km from Montpellier zoo, also in the south of France. We identified two more animals with antibodies against USUV confirming a relatively high prevalence for USUV in this species (33.3%, 4/12). 

Among all mammalians and more particularly in the order *Carnivora*, *Canidae* presented the highest USUV seroprevalence as we detected 25% (CI95%: 7.67–42.32) of positive specimens (Table A2).

To conclude, in this study statistically significant associations between seropositivity and sex or age were not observed. Among the species for which a relatively large sampling was available, Greater rhea (10/21) and to a lesser extent African wild dogs (4/12) had the highest infection rates. Blood samples from species showing high seroprevalence were additionally analyzed by RT-PCR for the presence of USUV or WNV RNA but we did not identify acute viral infection in these available samples. 

## 3. Discussion

Among arboviruses, WNV and USUV are neurotropic viruses characterized by an overlapping geographic distribution, host and vector ranges, as well as similar clinical manifestations [7]. The epidemiology of both WNV and USUV in Europe has changed dramatically over the last two decades. Studies performed in Italy provided evidence that USUV circulation in humans seems to be higher than WNV infection in some endemic regions where both viruses circulate [3,12,37,38]. At the end of the 90 s, there have been several reports of WNV outbreaks in Europe. Subsequently, this virus has spread widely in Central and Southern European countries, causing more important epidemics [39,40,41,42,43,44,45,46]. In 2018, WNV infections in Europe increased considerably compared to previous transmission seasons causing the biggest epidemic ever listed on the European continent [26]. This epidemic has affected more people in one year than during the previous 10 years across Europe and was concomitantly associated with a major USUV epidemic affecting several European countries and causing massive mortality in several bird species [3,28,47,48]. In accordance with human epidemiological data, our results suggest that USUV, and to a lesser extend WNV, may have circulated in zoo animals between 2016 and 2018 given that the two-thirds of seropositive animals analyzed were infected between 2016 and 2018. This higher seroprevalence during this period supports observations of an intense transmission in the surveyed area in the 2016–2018 period compared to previous transmission seasons. This exceptional level of viral circulation in 2018 for both USUV and WNV is probably related to several environmental and ecological factors influencing the spreading of these viruses such as elevated temperatures and precipitation anomalies, and other environmental factors that can favor mosquito breading [49,50].

Wild birds play a major role as reservoir hosts and as carriers for zoonotic arboviruses and their arthropod hosts to Europe. Every year, millions of birds migrate between Europe and Africa and play an important role in the spread of viruses to new areas. Both WNV and USUV can commonly infect at least 36 species of birds. Studying the seroprevalence of WNV and USUV in the avian population from zoos is therefore a good way to monitor the circulation of these viruses notably linked to the greater awareness of avian mortality in zoos compared to wild avifauna. Moreover, zoos are places where humans, mosquitoes, and birds are gathered in a relatively small space. Birds from zoological gardens have been described as victims of USUV infection. The first major outbreaks of USUV was identified in 2001 with the death of five great grey owls (*Strix nebulosa*) in a Vienna zoo [34]. In 2006 massive death were also reported in wild and captive avian species members of the orders Passeriformes (blackbirds and sparrows) and Strigiformes (owls) at a Zurich zoo [34] and deaths were reported in captive owls in Italy the same year [51]. USUV seroprevalence was studied in the zoos of Vienna, Zurich, and Basel with 5.3% to 8.75% of birds showing USUV-specific antibodies by PRNT-90, whereas no USUV antibodies were detected in the birds of the Budapest zoo [32]. Moreover, one of the first recorded WNV outbreaks in America occurred at the Bronx zoo in 1999 [52]. We identified several new avian species that appear to be susceptible to infection by WNV and USUV. Moreover, the seroprevalence appeared to be more important for USUV than for WNV in the Montpellier zoo. Of note USUV infections in the same area had previously been reported in mosquitoes [53], birds [54], and human [55] whereas WNV was reported in birds [54], horses [56,57,58], and humans [57,59,60]. One of the surprising outcomes of this study was that among USUV positive samples we found a very high seroprevalence for common peafowls (25.0%), emus (40.0%), and greater rheas (47.6%). To the best of our knowledge, these species have never been described as susceptible to flavivirus infection. Interestingly we identified four new orders susceptible to USUV infection (*Casuariiformes*, *Pelecaniformes*, *Psittaciformes*, and *Rheiformes*) and in particular we detected a very high seroprevalence for *Casuariiformes* and *Rheiformes* orders (40.0% and 47.6% respectively). At this point, the higher prevalence observed in *Casuariiformes* and *Rheiformes* could be due to either a higher susceptibility to flavivirus infection or to a higher rate of vector feeding (for example, due to their large size). Montpellier zoo has abundant populations of *Culex pipiens*, the main mosquito vector of USUV and WNV in the area (S. Gutierrez, personal communication). Studies on the blood-meal preference in these populations could help to define if those vertebrate species are preferred by mosquito vectors over other vertebrates in the zoo. In our study the detection of stable antibodies titers is probably associated with either the persistence of antibodies for a long time as previously described for USUV and WNV or to periodic circulation of these viruses in the area [29,30].

Interestingly we found no flavivirus antibodies in non-human primates in the 27 individuals from the nine species that we studied (Alouatta caraya, Callithix jacchus tibetana, Eulemur coronatus, Eulemur mongoz, Hapalemur alaotrensis, Lemur catta, Pithecia pithecia, Saguinus imperator, and Varecia rubra). Although these observations must be interpreted with caution due to the limited number of animals it seems that primates are not particularly sensitive to infections by USUV and WNV may be due to the feeding preferences of *Culex pipiens* for avian species. Another study previously described a low prevalence in primates from several zoological gardens [32]. On the other hand, unlike what has been found previously in another zoo, we did not detect flavivirus antibodies in the order Perissodactyla and especially in the Equidae family in the 18 animals studied although horses have been shown to be animals particularly susceptible to USUV and WNV [22,32,61,62,63]. Interestingly we found a high seropositivity for USUV in Carnivora, especially in Canidae, as previously described mainly in studies on domestic dogs [23,24,64]. Our results show that other Canidae than dogs could be potentially used as sentinel animals for virus surveillance especially since a longer exposure to arboviruses can be inferred from the fact that they spend most of the time outside. Free-living mammals are naturally exposed to flavivirus infections and, thus additional studies on their host competence would help in determining if they could be involved in the virus transmission cycle.

To conclude, our study shows a relatively high exposure of some species of exotic birds and mammals to USUV and to a lesser extent to WNV in a particularly exposed area of France. The overall seroprevalence was 0.73% for WNV and 6.57% for USUV. Our results correlate with a circulation of these two viruses reported in particular in 2018. This study highlights the value of the interest developing monitoring programs to better prevent, detect, and alert arboviruses circulation in areas gathering a large numbers of animals, humans, and vectors such as zoological gardens. 

## 4. Materials and Methods

### 4.1. Animal Samples

Montpellier zoo (south of France) covers an area of 80 hectares and is surrounded by an urban area and host some 106 animal species including birds and mammals. The sera were collected in this zoo between 2003 and 2019. In total, the sera of 411 animals belonging to 70 species and to 18 different orders were analyzed (for birds: *Anseriformes*, *Casuariiformes*, *Ciconiiforme*, *Coraciiformes*, *Galliformes*, *Gruiformes*, *Musophagiformes*, *Pelecaniformes*, *Psittaciformes*, *Rheiformes*, and *Struthioniformes*, for mammals: *Artiodactyla*, *Carnivora*, *Diprotodontia*, *Perissodactyla*, *Pilosa*, *Primates*, and *Rodentia*). Among them, samples from 137 zoo-birds, belonging to 20 different species and from 274 mammals from 50 different species were investigated. Samples originate from serum banks and animals undergoing medical check-ups. For all samples, 0.3 to 3 mL of blood was drawn from the cutaneous ulnar vein, the jugular vein, or a femoral vein. The blood was centrifuged at 2000× *g* for 10 min, the serum was then separated from the clot, and stored at −20 °C until analysis. Experiments were performed according to national regulations. The collection of samples was performed by veterinarians of the zoo. Samples were collected from serum banks or from animals subjected to sanitary check-ups, health programs, or surgical interventions. No animal was sampled for the only purpose of this study.

### 4.2. cELISA

Sera were screened using a cELISA test (ID Screen West Nile Competition Multi species ELISA kit, (IDVet, France). The technique is based on plates pre-coated with the envelope (E) protein of WNV and measure the competition between antibodies present in the animal serum tested and a monoclonal anti-WNV.E antibody conjugated to horseradish peroxidase (HRP). This test produces cross-reactions notably with other flaviruses and can therefore detect a large spectrum of flaviviruses including WNV and USUV and other related flaviviruses [65,66]. Assays were performed and interpreted according manufacturer instructions as previously described [66]. 

### 4.3. Seroneutralization Assays

The MNT was carried out in flat-bottomed 96 well microplates after serum heat-inactivation at 56 °C for 30 min. First 50 μL of Dulbecco’s modified Eagle’s (DMEM) medium (Thermoscientific) supplemented by 2% heat-inactivated fetal bovine serum (Sigma) was added in each well. Serum was subsequently added to a well in the first column to obtain a starting dilution of 1:5. From this well, two-fold dilutions were made by pipetting 50 μL of each well in the next, until the last column. The sera were mixed with 50 μL of virus suspension (at 100 tissue culture infectious dose 50 (TCID_50_) of USUV (France2018, MT863562) or WNV (lineage 2, MT863560) or TBEV (strain Hypr, Genbank ID U39292.1) or MEAV (Brest ART707, Genbank ID AF013386.1, DQ235144)), incubated at 37 °C for 90 min to allow neutralization of the virus. Wells were then supplemented with 100 μL of Vero E6 cell suspension (ATCC) (DMEM with 2% fetal bovine serum) and incubated 3 to 5 days at 37 °C and under 5% CO_2_ following the virus. After 3 days (WNV, USUV) and 5 days (TBEV, MEAV) microplates were read under the microscope to determine the presence or absence of cytopathogenic effect in samples and in positive and negative sera controls. In regards to MEAV, PRNT90 assays with sera diluted 1:20 was performed on SW13 cells as previously described [67]. Each sample was assigned a titer that was the reciprocal of the dilution in that well. Sera that showed neutralization at dilutions ≥1:10 (WNV, USUV) and ≥1:20 (TBEV, MEAV) were considered positive. When neutralization was observed against WNV and USUV, the neutralizing immune response was considered specific if the VNT titer for any given virus was at least fourfold higher than titers obtained against the other viruses. Duplicates were performed to confirm positive results.

### 4.4. RT-qPCR

Viral RNAs were extracted from 50 μL of serum with the EZ1 apparatus running the EZ1 DSP virus kit (Qiagen). Viral RNA levels were measured by a one-step quantitative reverse transcriptase PCR assay (RT-qPCR) on the Light Cycler 480 (Roche) with primers, probe, and cycling conditions previously described for USUV [68] and WNV [69].

### 4.5. Statistical Analysis

Seroprevalence for USUV and WNV was estimated by dividing the number of positive animals by total animals tested, using two-sided exact binomial 95% confidence intervals (95%CI). Correlation between the seroprevalence and other independent variables (sex, age, etc.) were analyzed by means of a Pearson’s chi-square test or by the Fisher’s exact test (less than 6 samples per group).

## Figures and Tables

**Table 1:** Birds species tested by ELISA (enzyme linked immunoabsorbent assay) and for ELISA-positive samples by MNT (microneutralization tests) for West Nile virus (WNV) and Usutu virus (USUV).

Avian Species							
Species	Species	Collection	Nb of		MNT Antibodies Titer		
(English Name)	(Scientific Name)	Date	Specimens	cElisa	USUV	WNV	Conclusion
Orange-winged amazon	*Amazona amazonica*	2016–2018	8	1	Neg	10	1/8 WNV
Brazilian teal	*Amazonetta brasiliensis*	2016–2018	6	0	Neg	Neg	Neg
Buffon’s macaw	*Ara macao*	2016–2018	2	0	Neg	Neg	Neg
Great green macaw	*Ara ambiguus*	2016–2018	4	0	Neg	Neg	Neg
White-winged duck	*Asarcornis scutulata*	2016	2	0	Neg	Neg	Neg
Abyssinian ground hornbill	*Bucorvus abyssinicus*	2016–2018	5	1	640	20	1/5 USUV
Greater vasa parrot	*Coracopsis vasa*	2019	2	0	Neg	Neg	Neg
Southern screamer	*Chauna torquata*	2016–2018	2	0	Neg	Neg	Neg
White-faced whistling duck	*Dendrocygna viduata*	2016–2018	1	0	Neg	Neg	Neg
Emu	*Dromaius novaehollandiae*	2016–2018	5	2	160	20	2/5 USUV
Scarlet ibis	*Eudocimus ruber*	2016–2018	38	1	640	10	1/38 USUV
Brahma chicken	*Gallus domesticus brahma*	2016	4	0	Neg	Neg	Neg
Marabou stork	*Leptoptilos crumenifer*	2016–2018	3	1	10	40	1/3 WNV
Blue-crowned motmot	*Momotus momota*	2019	1	0	Neg	Neg	Neg
Common peafowl	*Pavo cristatus*	2016–2018	24	6	10	Neg	6/24 USUV
					10	Neg	
					10	Neg	
					20	Neg	
					40	Neg	
					40	Neg	
Grey-winged trumpeter	*Psophia crepitans*	2019	2	0	Neg	Neg	Neg
Greater rhea	*Rhea americana*	2016–2018	21	10	640	10	10/21 USUV
					640	Neg	
					320	10	
					640	10	
					320	Neg	
					320	Neg	
					640	10	
					640	10	
					40	10	
					20	Neg	
African comb duck	*Sarkidiornis melanotos*	2016–2018	3	0	Neg	Neg	Neg
Common ostrich	*Struthio camelus*	200–20016	3	1	Neg	Neg	Other flavivirus
Red-crested turaco	*Tauraco erythrolophus*	2015	1	0	Neg	Neg	

Legend: In seropositive birds, titers for USUV and WNV are indicated. Titer was considered specific when MNT titer was at least fourfold higher against one virus compared to the other.

**Table 2:** Mammals species tested by ELISA and by MNT for ELISA-positive samples.

Mammal Species							
Species	Species	Collection Date	Nb of Specimens	MNT Antibodies Titer			Conclusion
(English Name)	(Scientific Name)			cELISA	USUV	WNV	
Cheetah	*Acinonyx jubatus*	2016–2018	6	1	Neg	Neg	Other flavivirus
Addax	*Addax nasomaculatus*	2015–2019	12	Neg	Neg	Neg	Neg
Black howler	*Alouatta caraya*	2015–2018	3	Neg	Neg	Neg	Neg
Barbary sheep	*Ammotragus lervia*	2014–2019	10	Neg	Neg	Neg	Neg
Hog deer	*Axis porcinus*	2016–2018	5	Neg	Neg	Neg	Neg
Sichuan takin	*Budorcas taxicolor tibetana*	2015	1	Neg	Neg	Neg	Neg
Common marmoset	*Callithrix jacchus*	2017	1	Neg	Neg	Neg	Neg
Dromedary	*Camelus dromedarius*	2016–2018	2	1	Neg	Neg	Other flavivirus
Iberian wolf	*Canis lupus signatus*	2013–2018	10	1	40	Neg	1/10 USUV
White rhinoceros	*Ceratotherium simum*	2004–2019	5	Neg	Neg	Neg	Neg
Sika deer	*Cervus nippon*	2016	8	Neg	Neg	Neg	Neg
Linnaeus’s two-toed	*Choloepus didactylus*	2016	2	Neg	Neg	Neg	Neg
sloth							
Maned wolf	*Chrysocyon brachyurus*	2015–2018	2	1	40	Neg	1/2 USUV
Prehensile-tailed	*Coendou prehensilis*	2016	2	Neg	Neg	Neg	Neg
porcupine							
Lowland paca	*Cuniculus paca*	2017	3	Neg	Neg	Neg	Neg
Blesbok	*Damaliscus pygargus*	2017	1	Neg	Neg	Neg	Neg
	*phillipsi*						
Somali wild ass	*Equus africanus*	2004	2	Neg	Neg	Neg	Neg
	*somaliensis*						
Grévy’s zebra	*Equus grevyi*	2017	2	Neg	Neg	Neg	Neg
Persian onager	*Equus hemionus onager*	2004–2016	5	Neg	Neg	Neg	Neg
Hartmann’s	*Equus zebra hartmannae*	2017–2019	4	Neg	Neg	Neg	Neg
mountain zebra							
Crowned lemur	*Eulemur coronatus*	2017–2018	4	Neg	Neg	Neg	Neg
Mongoose lemur	*Eulemur mongoz*	2016–2017	2	Neg	Neg	Neg	Neg
Rothschild’s giraffe	*Giraffa camelopardalis*	2015	1	Neg	Neg	Neg	Neg
	*rothschildi*						
Alaotran gentle	*Hapalemur alaotrensis*	2016–2018	7	Neg	Neg	Neg	Neg
lemur							
Sable antelope	*Hippotragus niger*	2017	4	Neg	Neg	Neg	Neg
Nile lechwe	*Kobus megaceros*	2018–2019	4	Neg	Neg	Neg	Neg
Ring-tailed lemur	*Lemur catta*	2017	5	Neg	Neg	Neg	Neg
Northern tiger cat	*Leopardus tigrinus*	2018	1	Neg	Neg	Neg	Neg
African wild dog	*Lycaon pictus*	2016–2019	4 ^a^ + 8 ^b^	2 ^a^ + 2 ^b^	40	Neg	4/12 USUV
					160	Neg	
					40 ^a^	Neg ^a^	
					40 ^a^	Neg ^a^	
Bennett’s wallaby	*Macropus rufogriseus*	2016–2018	12	Neg	Neg	Neg	Neg
Kirk’s dik-dik	*Madoqua kirkii*	2016–2019	4	Neg	Neg	Neg	Neg
Reeves’s muntjac	*Muntiacus reevesi*	2017	4	Neg	Neg	Neg	Neg
Giant anteater	*Myrmecophaga tridactyla*	2018	2	Neg	Neg	Neg	Neg
Dama gazelle	*Nanger dama*	2017–2019	14	1	Neg	10	1/14 WNV
Beisa oryx	*Oryx beisa*	2017	6	Neg	Neg	Neg	Neg
Scimitar-horned oryx	*Oryx dammah*	2016–2018	2	Neg	Neg	Neg	Neg
Arabian oryx	*Oryx leucoryx*	2016–2019	6	Neg	Neg	Neg	Neg
Caussenarde Sheep	*Ovis aries*	2016–2019	34	Neg	Neg	Neg	Neg
Cameroon Sheep	*Ovis aries cameroon*	2018–2019	4	Neg	Neg	Neg	Neg
Atlas lion	*Panthera leo leo*	2003–2019	4	1	Neg	Neg	Other flavivirus
Asian lion	*Panthera leo persica*	2017	1	1	40	Neg	1/1 USUV
White-faced saki	*Pithecia pithecia*	2018	1	Neg	Neg	Neg	Neg
Bharal	*Pseudois nayaur*	2015–2019	26	Neg	Neg	Neg	Neg
Emperor tamarin	*Saguinus imperator*	2019	3	Neg	Neg	Neg	Neg
South American tapir	*Tapirus terrestris*	2017	1	Neg	Neg	Neg	Neg
Nyala	*Tragelaphus angasii*	2016–2019	4	Neg	Neg	Neg	Neg
Eastern bongo	Tragelaphus eurycerus	2017–2019	3	Neg	Neg	Neg	Neg
	isaaci						
Common eland	*Tragelaphus oryx*	2016–2019	5	Neg	Neg	Neg	Neg
Lesser kudu	*Tragelaphus imberbis*	2016	2	Neg	Neg	Neg	Neg
Syrian brown bear	*Ursus arctos syriacus*	2015–2017	3	Neg	Neg	Neg	Neg
Red ruffed lemur	*Varecia rubra*	2018–2019	2	Neg	Neg	Neg	Neg
Vicuna	*Vicugna vicugna*	2017–2019	5	Neg	Neg	Neg	Neg

Legend: ^a^—Montpellier zoo; ^b^—Sigean zoo.

## Appendix A

**Table A1:** WNV and USUV seroprevalence in longitudinally sampled birds (2016 and 2018).

	2016		2018		
Species	USUV	WNV	USUV	WNV	Period of Infection
*Amazona amazonica*	Neg	Neg	Neg	10	between 2016 and 2018
*Dromaius novaehollandiae*	80	10	160	10	before 2016
*Eudocimus ruber*	Neg	Neg	640	10	between 2016 and 2018
*Leptoptilos crumenifer*	10	20	10	40	before 2016
*Pavo cristatus*	Neg	Neg	40	10	between 2016 and 2018
	10	Neg	10	Neg	before 2016
*Rhea americana*	640	20	640	20	before 2016
	Neg	Neg	640	Neg	between 2016 and 2018
	20	Neg	320	10	before 2016
	Neg	Neg	640	10	between 2016 and 2018
	Neg	Neg	320	Neg	between 2016 and 2018
	Neg	Neg	320	Neg	between 2016 and 2018
	Neg	Neg	640	10	between 2016 and 2018
	Neg	Neg	640	10	between 2016 and 2018

**Table A2:** Prevalence of WNV or USUV antibodies in birds and mammalian species sampled classified by order and family.

Classification	Number	Positives	%WNV	%USUV
**Class Aves Order Anseriformes**				
Family Anatidae	12	0	0.0	0.0
Family Anhimidae	2	0	0.0	0.0
**Order Casuariiformes**				
Family Casuariidae	5	2 USUV	0.0	40.0
**Order Ciconiiforme**				
Family Ciconiidae	3	1 WNV	33.0	0.0
**Order Coraciiformes**				
Family Momotidae	1	0	0.0	0.0
**Order Galliformes**				
Family Phasianidae	28	6 USUV	0.0	17.2
**Order Gruiformes**				
Family Psophiidae	2	0	0.0	0.0
**Order Musophagiformes**				
Family Musophagidae	1	0	0.0	0.0
**Order Pelecaniformes**				
Family Threskiornithidae	38	1 USUV	0.0	2.6
**Order Psittaciformes**				
Family Psittacidae	16	1 WNV	6.2	0.0
Family Psittaculidae	5	1 USUV	0.0	20.0
**Order Rheiformes**				
Family Rheidae	21	10 USUV	0.0	47.6
**Order Struthioniformes**				
Family Struthionidae	3	0	0.0	0.0
**TOTAL Birds**	**137**	**22**	**1.45**	**14.59**
**Class Mammalia Order Artiodactyla**				
Family Bovidae	142	1 WNV	0.7	0.0
Family Cervidae	17	0	0.0	0.0
Family Camelidae	7	0	0.0	0.0
Family Giraffidae	1	0	0.0	0.0
**Order Carnivora**				
Family Canidae	24	6 USUV	0.0	25.0
Family Felidae	12	1 USUV	0.0	8.3
Family Ursidae	3	0	0.0	0.0
**Order Diprotodontia**				
Family Macropodidae	12	0	0.0	0.0
**Order Perissodactyla**				
Family Equidae	13	0	0.0	0.0
Family Rhinocerotidae	5	0	0.0	0.0
Family Tapiridae	1	0	0.0	0.0
**Order Pilosa**				
Family Choloepodidae	2	0	0.0	0.0
Family Myrmecophagidae	2	0	0.0	0.0
**Order Primates**				
Family Atelidae	3	0	0.0	0.0
Family Callitrichidae	4	0	0.0	0.0
Family Lemuridae	20	0	0.0	0.0
Family Pitheciidae	1	0	0.0	0.0
**Order Rodentia**				
Family Cuniculidae	3	0	0.0	0.0
Family Erethizontidae	2	0	0.0	0.0
**TOTAL Mammalian**	**274**	**8**	**0.36**	**2.55**
**TOTAL Animals**	**411**	**30**	**0.73**	**6.57**
